# Nanocellulose-Based Biomedical Scaffolds in Future Bioeconomy: A Techno-Legal Assessment of the State-of-the-Art

**DOI:** 10.3389/fbioe.2021.789603

**Published:** 2022-02-11

**Authors:** Pawan Kumar Mishra, Ondrej Pavelek, Martina Rasticova, Harshita Mishra, Adam Ekielski

**Affiliations:** ^1^ Faculty of Business and Economics, Mendel University in Brno, Brno, Czechia; ^2^ Smart Society Research Team, Faculty of Business and Economics, Mendel University in Brno, Brno, Czechia; ^3^ Department of Production Engineering, Warsaw University Of Life Sciences, Warsaw, Poland

**Keywords:** Nanocellulose, Cellulose, REACh regulation, EMA, Bioeconomy, Biomedicine

## Abstract

Nanocellulose is a broader term used for nano-scaled cellulosic crystal and/or fibrils of plant or animal origin. Where bacterial nanocellulose was immediately accepted in biomedicine due to its “cleaner” nature, the plant-based nanocellulose has seen several roadblocks. This manuscript assesses the technological aspects (chemistry of cellulose, nanocellulose producing methods, its purity, and biological properties including toxicity and suggested applications in final drug formulation) along with legal aspects in REACH (Registration, Evaluation, Authorization, and Restriction of Chemicals) regulation by the European Union, EMA (European Medicine Agency). The botanical biomass processing methods leading to the nanoscale impurity (lignin and others) on nanocellulose surface, along with surface modification with harsh acid treatments are found to be two major sources of “impurity” in botanical biomass derived nanocellulose. The status of nanocellulose under the light of REACH regulation along with EMA has been covered. The provided information can be directly used by material and biomedical scientists while developing new nanocellulose production strategies as well as formulation design for European markets.

## Introduction

Nanocellulose or nano-structured cellulose is general term used for cellulose fibers with diameters around 5–20 nm and varying degree of length (depending on source and processing parameters). Generally, nanocellulose includes three categories of cellulose, which are 1) Cellulose Nano Fibrils (CNF)/Nano Fibrillated Cellulose (NFC), 2) Cellulose Nano Crystals (CNC)/Cellulose Nano Whiskers (CNWs), and 3) Bacterial Nano Cellulose (nano structured cellulose of bacterial origin, BNC) (Klemm et al., 2011).

**FIGURE 1:**
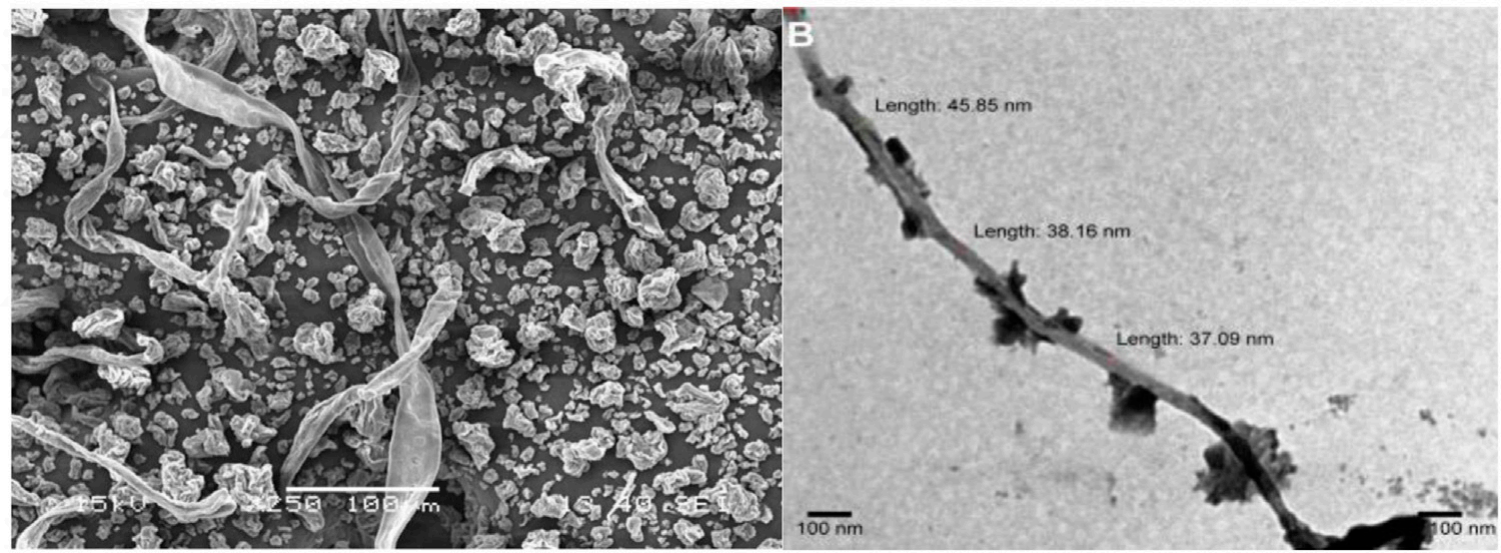
Electron microscopic structure of nanocellulose (Bhandari et al., 2017; Mishra et al., 2017, 2019).

The classical approach to synthesize CNC is by acid hydrolysis (mostly sulfuric acid; phosphoric acid and hydrochloric acid are also reported) (Abitbol et al., 2016). Recently, two new methods have also been patented by the companies American Process Inc. (Nelson et al., 2014) and Blue Goose Biorefineries Inc. (Olkowski and Laarveld, 2013). These two methods were based on acid and solvent-based pretreatment to minimize mechanical energy consumption and transition metal-based nano-catalyst for biorefinery, respectively. CNF is mainly produced by mechanical processing assisted by enzymatic or chemical pretreatments. Mechanical methods to produce CNF include high pressure homogenization (HPH), microfluidization, grinding, cryocrushing, and high intensity ultrasonication (Abdul Khalil et al., 2014). HPH involves passing of cellulose slurry through a fine nozzle into a vessel. Size reduction takes place mainly due to shear forces generated at high pressure, velocity, and impact forces (Figure 1). First reports of the HPH process are as old as 1983 (Herrick et al., 1983). Microfluidizer is similar to HPH with an additional pump to generate high pressure streams. Interaction chamber here collides the streams and walls to create the fibrillating forces (Ferrer et al., 2012). Grinding involves passage of cellulose slurry between the static and mobile grinding stones, and motion of the stone provides the fibrillating force to get nanoscale fibers (Wang et al., 2012). In cryocrushing, water swollen cellulosic fibers frozen by liquid nitrogen are crushed using a mortar and pestle. The crushing impact forces and force exerted by ice crystals during the process create the liberating force for cellulose fibers from the cell wall of plant materials (Siró and Plackett, 2010). During high intensity ultrasonication, cavitation leads to powerful mechanical oscillating power involving formation, expansion, and implosion of microscopic gas bubbles due to absorption of ultrasonic energy by molecules (Chen et al., 2013).

Bacterial nanocellulose or biocellulose is the cellulose synthesized by the action of microorganisms. It is synthesized mainly by the bacterium *Gluconacetobacter xylinus* (also named as *Acetobacter xylinus*). Some other microorganisms also exhibit the ability to synthesize the cellulose, such as other species of *Gluconacetobacter*, *Agrobacterium tumefaciens*, *Rhizobium spp*., and Gram-positive *Sarcina ventriculli* (Tanskul et al., 2013; Mohammadkazemi et al., 2015; Jozala et al., 2016). *G. xylinus* is the primary microbe producing the biocellulose and commonly studied as a model system for the study of the biosynthetic mechanisms of cellulose synthesis. For the production of cellulose, *G. xylinus* secretes a nanofibrillar film with a denser lateral surface and a gelatinous layer on the opposite side (Kurosumi et al., 2009). The process of cellulose synthesis by *G. xylinus* can be explained in three steps: 1) polymerization of glucose residues, 2) extracellular secretion of linear chains, and 3) organization and crystallization of glucan chains (from Step 1) in a hierarchy into fibrils/strips (Klemm et al., 2011).

In regard of the established applications of the above three varieties of nanocelluloses, bacterial nanocellulose based biomedical products (wound care and wound healing) are already being marketed. Botanical-biomass based celluloses are mainly used in reinforcement and composite applications. There are several reasons for easy acceptability of bacterial nanocellulose in biomedical applications. First is its inherent purity. It is free of lignin, hemicelluloses, pectin, and other plant-based phenolic compounds (Chawla et al., 2009), and therefore it is simpler to purify as compared to botanical-NC. Second, bacterial-NC possesses a highly porous structure with high water absorption capacity that can help in absorbing exudates from wounds (Ullah et al., 2016). Finally, the lack of impurity provides a higher number of hydroxyl groups for surface modification, harmless degradation products (only glucose), negligible amount of endotoxin (approved by FDA for surgical sheets and tissue reinforcements), higher complement activation parameters as compared to alternates, slower blood-coagulation in comparison to clinically available materials, and lack of skin irritation potential (Fink et al., 2011; Petersen and Gatenholm, 2011; Almeida et al., 2014) and hence improved compatibility properties. On the other hand, the botanical-biomass based nanocellulose carry remnants of chemicals from processing steps, which can be explained as a main reason for their less acceptability in applications requiring a cleaner surface. Although new reports of botanical-biomass based nanocellulose drug delivery and wound care system are showing promising results, this area needs more reports and tests to reach a reasonably strong conclusion.

## Chemistry, Crystallinity, and Biological Properties of Cellulose

Cellulose is a linear polymer of D-glucopyranose units linked by β-1,4-glycosidic bonds. At molecular levels, it is composed of carbon (44.44%), hydrogen (6.17%), and oxygen (49.39%). Cellulose can also be represented by a basic chemical formula of (C_6_H_10_O_5_)n; where n is called as the degree of polymerization (DP), attributed to the number of glucose groups, ranging from hundreds to thousands, varying from source to source and also affected by the method of processing for obtaining the cellulose. Traditionally, β-cellulose and γ-cellulose (Table 1) constitute industrial hemicellulose, and holocellulose refers to all the carbohydrates (cellulose and hemicellulose).

**TABLE 1:** Classification of cellulose based on DP values (Staudinger, 1933).

Type of cellulose	Method	DP
α-cellulose	Insoluble in 16.5% NaOH at 20°C	>200
β-cellulose	Precipitated from above solution using acid for neutralization	10–200
γ-cellulose	Soluble in acidic, alkaline, and neutralized solution	<10

**FIGURE 2:**
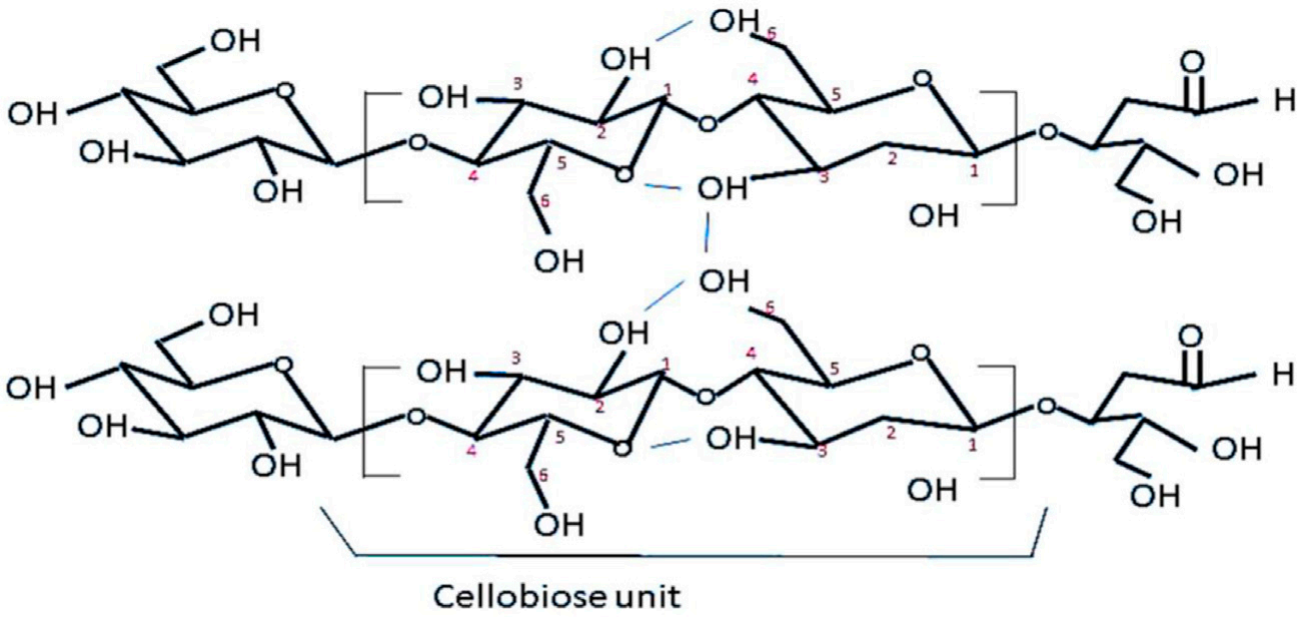
Chemical structure of cellulose with a numbering system for various positions.

In the chemical structure of cellulose (Figure 2), the primary structure (linear chain and stereo-chemical structure) shows a cellobiose unit, reducing end (open ring structure), and non-reducing end (close ring structure). Within the linear chain, every glucose unit can be seen rotated by 180° and new glucose units are added at non-reducing ends deciding the direction of chain growth. The intramolecular hydrogen bonding can be seen in hydroxyl groups attached to the 2nd and 6th carbons, hydroxyl groups attached to the 3rd carbon, and adjacent oxygen from adjacent molecules. The intramolecular hydrogen bonds and van der Waal forces come into play during stacking of cellulose chains one above the other, between hydroxyl groups attached to the 3rd and 6th carbons of stacking chains. Surface characteristics of cellulose are most important for surface modifications, which in turn play a pivotal role in final product applications. Starting from the molecular level, each anhydro-glucopyranose unit does not lie in the plane of their structure but they assume a chair form (lowest energy conformational isomer) and each next molecule is rotated by 180° about the molecular axis with hydroxyl group at the equatorial position (Habibi et al., 2010). Each cellulose unit has three hydroxyl groups in cellulose, at the 2nd, 3^rd^, and 6th positions (Figure 2). At the 6th position, the hydroxyl group behaves as a primary carbon but at the 2nd and 3rd positions it acts as a secondary alcohol. Comparing the reactivity, the hydroxyl group at the 6th position shows 10 times more reactivity than the other two and the hydroxyl group at the 2nd position is twice as reactive as that at the 3rd position.

However, in the case of surface characteristics of nanocellulose, the chemical nature of the reactive agents and solvents also plays a major role in determination of reactivity. The reactivity of the hydroxyl group toward the nucleophilic attack was reported as almost the same for the 6th and 2nd carbon, which is more than reactivity of the hydroxyl group at the 3rd carbon (de la Motte et al., 2011; Lin and Dufresne, 2014).

In the specific case of CNC, it is produced by hydrolysis of an amorphous region of cellulose using sulfuric acid, leaving behind the crystalline part. This process results in negatively charged sulfate esters (condensation esterification/sulfation) on the CNC surface, which determines most of the surface characteristics of resultant material. As the surface charge is mainly due to sulphate esters, it is affected by duration and temperature of sulfation reaction (hydrolytic reaction).

Several methods based on XRD and ^13^C NMR spectra have been reported to study the crystallinity of cellulose. Based on XRD data, there can be a peak height method (Segal et al., 1959), peak deconvolution method (Hult et al., 2003; Garvey et al., 2005; He et al., 2008), and amorphous subtraction method (Ruland, 1961). In the NMR-based method used is C_4_ peak separation, the peak height method was based on a ratio of heights of 002 peak and minimum between 002 and 101 peak. This method worked well for comparative studies but had several limitations, too. Limitations include underestimation of a minimum due to its non-alignment with amorphous peak (Park et al., 2010); due to choice of only peak one out of at least four peaks, one specific orientation gets more attention than others; peaks in the cellulose spectrum are very wide and peak height cannot be used as an exact measure (Garvey et al., 2005). The amorphous subtraction method involves subtracting the amorphous spectra from diffraction pattern and crystallinity index (CI) is calculated as the ratio of crystalline area and total area. In this case, the challenge was to choose the right amorphous standard. Peak deconvolution method requires a software to separate different peaks. Four to five peaks have been separated in different cases. This method assumes an amorphous component as the main contributor to peak broadening, but other factors like crystalline size and non-uniform strain with the sample can also lead to the same results. Another aspect is that cellulose peaks are very broad and are resolved only with peak overlaps.

In the ^13^C NMR method, the ratio of the area under C_4_ peaks (assigned to crystalline cellulose at 89 ppm) and total areas of C_4_ peaks (assigned to amorphous cellulose at 84 ppm and crystalline peak both) are considered as CI values. Based on ^13^C CP/MASS NMR spectra reports in which C (4) and C (6) of cellulose show different signals (Horii et al., 1982), cellulose polymorphs can be divided into two groups: Cellulose (I, III_I_, and IV_I_) and Cellulose (II, III_II_, and IV_II_). Cellulose I (the native cellulose) has cellulose chains arranged in such a way that glucopyranose rings are parallel to the “bc” plane of crystal (Gardner and Blackwell, 1974), hence exposing the glucopyranose ring from one crystal to another one. This property has been supported by evidence of binding enzymes on glucopyranose structures. The native cellulose (cellulose I) also shows two polymorphs called I_α_ and I_β_. The former is dominant in cellulose produced by primitive organisms whereas the latter dominates in higher plants. I_α_ has triclinic one chain unit cell in which chains are stacked by van der Waal forces and I_β_ has monoclinic two chain unit cells stacking with alternating shear. The I_α_ gets converted into I_β_ upon hydrothermal treatment and some solvents. Cellulose II is the result of alkali treatment of cellulose I. Cellulose II has cellulose chains rotated by 30° from the parallel to “ab” crystal phase (most applicable for 2 chain model but some cellulose requires 8 chain models). Additionally, intermolecular hydrogen bonding is significantly complex in crystalline parts of cellulose II as compared to cellulose I. The main difference lies in the configuration of the 6th carbon. For cellulose I this arrangement is *trans*-gauche (tg), whereas for cellulose II it is gauche-trans (gt) (Gardiner and Sarko, 1985). The additional feature of hydrogen bonding in cellulose II over cellulose I is due to intersheet bonding of C (2) hydroxyl group of corner chain and oxygen of the central chain, which is absent in native cellulose. Cellulose II has two chain monoclinic unit cells stacked by opposite polarity (antiparallel structure). Cellulose III_I_ and III_II_ can be reversibly transformed into cellulose I and II, respectively, using ammonia. Cellulose III_I_ is a monoclinic one chain unit cell where parallel cellulose chains are stacked with no stagger along the chain axis. Cellulose IV_I_ and IV_II_ are possible transformed products of cellulose I and II, respectively. Although some reports suggest that direct transformation of cellulose I and II to IV_I_ and IV_II_, respectively, does not happen in a step, there is possible formation of III_I_ and III_II_ (Wada et al., 2004). In the diffraction study of microfibrils, alternate light and dark regions have been found, which are explained as crystalline and amorphous cellulose, respectively. Amorphous cellulose has been explained by various postulates, including isotropically distributed straight chains (Fink et al., 1987), and bent and twisted chains (Paakkari et al., 1989). Furthermore, it was also postulated that light and dark bands could be because of a slight curvature which makes in and out of Bragg’s diffraction conditions (Gautam et al., 2010). More details on cellulose crystallinity are beyond the scope of this article. Authors recommend an excellent work on this topic by Park et al. (2010).

Cellulose does not get digested in the human gut due to the absence of β-1-4 glycosidic bond cleaving enzymes, which is present in ruminating animals. This property of cellulose is used for its utilization as excipient in drug delivery formulations. In the environment, microorganism degrading cellulose (called cellulases) produce two types of cellulases, namely, endoglucanases and cellobiohydrolases (CBH). Endoglucanases hydrolyze internal bonds (preferably the amorphous regions) releasing new terminal ends. CBH (exo-1,4-b-glucanases) act on the existing or endoglucanase-generated chain ends. Amorphous cellulose can be degraded by both types of enzymes, but crystalline cellulose is efficiently degraded only by CBH. Both cellulases release cellobiose molecules as the result of hydrolytic cleavage. Also, breakdown of cellobiose requires β-glucosidases, which converts it into two molecules of glucose. Further hydrolysis of glucose results in carbon and energy sources for cellulolytic microorganisms (Pérez et al., 2002).

Cellulose is relatively stable to UV absorption and decomposition as compared to lignin (Mishra and Wimmer, 2017). For the toxicity studies, each type of cellulose needs to be considered individually as the method of production affects the surface characteristics significantly, which in turn plays a significant role in toxicity. For purified cellulose, the production steps result in trace organochlorine contamination originating from the chemical reactions in the purification process. However, the chronic ingestion did not show any increase in spontaneous disease or neoplasia. Additionally, any promotional activity in the mammary gland, colon, or bladder of the rats was not reported and any negative impact on the absorption or the metabolism of dietary components was not concluded. Therefore, no adverse health effects in humans were suggested from exposure to purified cellulose (Anderson et al., 1992). For bacterial cellulose produced by *Gluconacetobacter xylinus,* Jeong et al. reported a study of toxicity of bacterial cellulose nanofibers in human umbilical vein endothelial cells (HUVECs) using viability and flow cytometric assays, and in C57/Bl6 mice. The absence of toxicity *in vitro* and *in vivo* supported the view that bacterial cellulose may be used as a tissue engineering biomaterial (Jeong et al., 2010). In a study done on respirable cellulose fibers, short-term inhalation of cellulose caused an inflammatory lung response which resolved despite continuing exposure. Intraperitoneal injection of cellulose fibers induced sarcomas rather than mesotheliomas at the highest dose (10^9^ fibers), while the two middle doses (10^7^ and 10^8^ fibers) each produced a mesothelioma (Cullen et al., 2002). In a review published on toxicity of CNC, it was mentioned that oral and dermal toxicity assessment of CNCs have shown a lack of adverse health effects, whereas studies on the pulmonary and cytotoxicity have yielded discordant results. Authors suggested the need for additional studies to support the general conclusion that CNCs are nontoxic on ingestion or contact with the skin and to determine whether CNCs have adverse health effects on inhalation or elicit inflammatory or oxidative stress responses at the cellular level.

## Biomedical Applications of Botanical Nanocellulose

Nanocellulose has gained great popularity as a drug carrier in the past few years. Owing to its several favorable characteristics, including nanosize, high surface area, bioavailability, biocompatibility, and surface tunable chemistry, different drug delivery systems could be explored. There have been hydrogels, gels, microparticles, membranes, scaffolds, and films. Not only the diversity in drug delivery systems, but also nanocellulose possesses diversity in route of administration. There are oral, transdermal, mucoadhesive, and even injectable formulations too. Further, nanocellulose has been used to deliver drugs locally, as well as systemically (Salimi et al., 2019).

One of the main applications of nanocellulose in drug delivery is the controlled and sustained release of the active pharmaceutical ingredient. Reportedly, nanocellulose sustains the drug release by forming a tight fiber network around the incorporated drug entities (Kolakovic et al., 2012). Also, the hydrogen bonding that nanocellulose forms with various drugs improves the stability and cohesion of the biopolymer matrix leading to a sustained release (Guo et al., 2017). Apart from controlled release of drugs, nanocellulose has served other purposes too in drug delivery, such as targeting, improved stability, better bioavailability, and increased solubility (Table 2). Nanocellulose can even be made to carry an imaging agent inside the body and thus aid in diagnostics (Colombo et al., 2015; Zhou et al., 2015).

**TABLE 2:** Biomedical applications of nanocellulose.

Formulation	Drugs	Application	Result
Drug delivery			
NCC	Tetracycline, doxorubicin (hydrophilic drugs)	Oral controlled release	NCC bound significant quantities of water soluble ionizable drugs, which were released rapidly over a 1-day period
			CTAB bound to the surface of NCC increased its zeta potential and could bind significant quantities of hydrophobic drugs, released in a controlled manner over a 2-day period
	Docetaxel, paclitaxel, etoposide (hydrophobic drugs)		Binding and uptake of NCC-CTAB nanocomplexes was observed in KU-7 cells (Burt et al., 2014)
NCC thin films along with chitosan	Doxorubicin hydrochloride (hydrophilic) curcumin (hydrophobic)	Oral controlled release	Major interactions of drugs with NCC were hydrogen bonding and van der Waals interactions
			Both drugs were released in a sustained manner
			Doxorubicin release in acidic conditions was higher due to protonation of the amine group that helped the diffusion process leading to a greater solubility. This is preferable for cancer treatment as pH of tumor cells is acidic (Mohanta et al., 2014)
NCC and micro crystalline cellulose (MCC)	Meloxicam	Increasing solubility	Co-grinding with MCC did not increase solubility
			Co-grinding with NCC increased the solubility up to 20% compared to ground drug powder, and more than 100% compared to initial unground powder, due to its nanodimension. Also, increasing NCC loading increased solubility and dissolution of meloxicam (Emara et al., 2016)
NFC-alginate and MCC-alginate beads	Metformin hydrochloride	Oral controlled release	MCC-alginate showed cumulative drug release of 56% in initial 60 min, followed by a rapid release
			NFC-alginate beads showed a sustained release over 240 min (Guo et al., 2017)
NFC nanocomposites - gelatinized maize starch and urea formaldehyde	Dimethyl phthalate (DMP)	Oral controlled release	NFC significantly hindered initial release of DMP while it improved overall release
			NFC networks within starch matrix caused a tortuous diffusion pathway for drug and prolonged the release (∼80–95% drug release over a week) (Patil et al., 2018)
Magnetic nanocellulose (*m*-NCC) alginate hydrogel beads	Ibuprofen	Oral controlled release	*m*-NCC incorporated into alginate beads could limit the movement of ibuprofen during dissolution because of their high physical involvement. Findings showed a controlled and sustained release of the drug (Supramaniam et al., 2018)
NFC-chitosan film	Ketorolec tromethamine	Transdermal controlled release	Sustained release profiles of the drug were observed from matrices. 40% drug was released in 10 h by addition of 1 wt% NFC in the formulation (Sarkar et al., 2017)
NFC	Indomethacin, itraconazole, beclomethasone	Transdermal controlled release	NFC was used to develop film-like matrix systems with drug loadings between 20 and 40%, and entrapment efficiency of >90%
			Drug release was sustained for 3 months with very close to zero-order kinetics (Kolakovic et al., 2012)
CNF-titania nanocomposites	Diclofenac sodium, penicillamine-D, phosphomycin	Transdermal controlled release	Formulations displayed distinctly different but controlled long-term release profiles
			Three different methods of medicine introduction showed various interactions between titania and drug molecules, and thus different kinetics of long-term drug release
			Conclusively, obtained nanocomposites could be used in transdermal drug delivery for anesthetics, analgesics, and antibiotics (Galkina et al., 2015)
Nanocellulose - metal nanocluster composite	Silver nanoclusters	Support material for transdermal delivery of antibiotic and antibacterial agents	NFC and MFC fibers were used as support for small and fluorescent silver nanoclusters
			Functionalization was mediated by poly (methacrylic acid) that protects nanoclusters while it allows hydrogen bonding with cellulose, leading to composites with fluorescence and antibacterial activity (Díez et al., 2011)
NFC hydrogel	123I-β-CIT (small drug)	Local	Hydrogel decreased the elimination rate of the large drug by twofold while retaining the release rate of the small drug
	99mTc-HAS (large protein)	Controlled release	Conclusively, NFC hydrogel is a promising matrix for controlled release or local delivery of large compounds (such as macromolecular protein and peptide drugs) (Laurén et al., 2014)
Anionic NFC, with mucoadhesive components (mucin, pectin, chitosan)	Metronidazole	Local mucoadhesive	Fast drug release was observed that can benefit the treatment of oral diseases, such as periodontitis (Laurén et al., 2018)
NFC - drug nanoparticles	Itroconazol	Improving storage stability	Nanofibrillar matrix of NFC provided protection to nanoparticles during formulation process and increased their storage stability
			In a suspension with NFC, drug nanoparticles could be stored for more than 10 months
			Dissolution rate of itraconazole was also increased thus improving its *in vivo* performance (Valo et al., 2011)
Pickering emulsion stabilized by aminated nanocellulose (ANC) particles	Coumarin, curcumin	Improving bioavailability and controlled release	Encapsulation efficiency of coumarin and curcumin were >90%
			Release kinetic profiles displayed sustained release with supposed increase bioavailability
			Studies performed on different microorganisms (Gram (+), Gram (−), and fungi) demonstrated the formulation as promising candidates to inhibit microbial growth (Asabuwa Ngwabebhoh et al., 2018)
CNC—folic acid	—	Targeting	Folate receptor mediated cellular uptake of conjugated CNC was demonstrated on human (DBTRG-05MG, H4) and rat (C6) brain tumor cells, and was 1,452, 975, and 46 times higher, respectively, than that of non-targeted CNC (Dong et al., 2014)
Chitosan oligosaccharide (CS_OS_) grafted on CNC	PrHy (model drug)	Local delivery	CNC–CS_OS_ nanoparticles showed binding efficiency of 21.5% and drug loading of 14% w/w
			Fast release of drug was observed in 1 h
			Conclusively, formulation can be used as fast response drug carriers in wound-dressings and local drug delivery to the oral cavity (Akhlaghi et al., 2013)
Tissue Engineering			
CNF - alginate hydrogels	—	Tissue repair and wound healing	Addition of CNF in alginate gels contributed to formation of porous structure and increased Ca^2+^ crosslinking density in gel structure
			CNF-alginate gels improved bioadhesion, growth, and proliferation of the cells onto the gels. (Siqueira et al., 2019)
Carboxymethylated NFC (c-NFC) biocomposite hydrogels	—	Tissue engineering	Biocomposite hydrogels could successfully mimic mechanical and swelling behavior of human nucleus pulposus (NP)
			Presence of c-NFC showed lower strain values after cyclic compression tests and consequently created improved material relaxation properties compared with neat hydrogels (Eyholzer et al., 2011)
CNF scaffold with CNCs embedded	—	Tissue engineering	Cells could proliferate rapidly on the surface and deep inside the formulation
			Aligned nanofibers exhibited strong effect on directing cellular organization, making the scaffold particularly useful for various artificial tissues or organs, such as blood vessel, tendon, and nerve, in which cell orientation is crucial (He et al., 2014)
NFC	—	Wound healing	NFC dressing was compared to commercial lactocapromer dressing, Suprathel^®^ (PMI Polymedics, Germany)
			Epithelialization of the NFC dressing-covered donor site was faster compared to Suprathel^®^
			NFC caused no allergic or inflammatory reaction (Hakkarainen et al., 2016)
Hemicellulose - NFC hydrogel scaffolds	—	Wound healing	Hemicellulose (galactoglucomannan, xyloglucan, and xylan) were introduced into NFC to reinforce NFC hydrogels
			Results revealed that all polysaccharide composite hydrogels may work as promising scaffolds in wound healing by providing supporting networks, and promoting cell adhesion, growth, and proliferation (Liu et al., 2016)
CNF	—	Wound healing	Growth curves using CNF in suspension demonstrated dose-dependent inhibition of bacterial growth
			Analysis of biofilm formation (P. aeruginosa PAO1) on nanocellulose aerogels revealed significantly less biofilm biomass (Jack et al., 2017)
Calcium ion - NFC hydrogels	—	Wound healing	NFC hydrogels presented entangled fibrous networks with solid-like behavior and were found to be inert in terms of cytokine secretion and ROS production
			These results establish ion-crosslinked NFC hydrogels as a good candidate for advanced wound dressings (Basu et al., 2017)
Biosensing and Bioimaging			
CNF functionalized with a natural pigment	Natural pigment from red cabbage	pH sensing	Developed biocomposite could detect pH values in the range of 1–14. pH sensing was stable at different temperatures and at prolonged times
			Colors were reversible and the pH sensor was recyclable
			This universal pH sensor can be used as a health monitor (Devarayan and Kim, 2015)
Cellulose nanocrystals	Quinolone fluorophore	Bioimaging	Formulation was used in interaction studies with carbohydrate-binding proteins, biorecognition, and in bacterial imaging
			Functional cellulose nanocrystals could selectively recognize cognate lectins
			Mannosylated nanocrystals selectively interacted with FimH-presenting E. coli (Zhou et al., 2015)
CNC	HNE tripeptide substrate	Biosensing, wound management	Elevated human neutrophil elastase (HNE) is a biomarker in chronic wounds
			HNE tripeptide substrate was covalently attached to glycine esterified CNC and compared with a similar tetrapeptide analog for colorimetric HNE sensor activity. Visible HNE activity was significantly higher on CNC tripeptide conjugates (Edwards et al., 2013)
CNC functionalized with fluorescent dye	Fluorescent dye	Theragnostic	Functionalized CNC could transiently migrate in bones and penetrate in the cytoplasm of cancer cells
			Interactions with bones was due to chemical interaction between Ca^(2+)^ of bone and negatively charged CNCs (Colombo et al., 2015)
Probe labeled CNF	Lucifer yellow derivative	Bioimaging	Luminescent CNF were exposed to live juvenile daphnids and microscopy analysis revealed the presence of the luminescent CNF all over D. magna’s alimentary canal tissues without any toxicity (Navarro et al., 2016)
Nanocellulose—carbon nanoparticles (NC-CNPs) nanocomposite film modified glassy carbon electrode (GCE)	—	Biosensing	Oxidation product of metoclopramide (an anti-emetic drug) gets adsorbed on the surface of NC-CNPs/GCE.
			Modified electrode showed a distinctive anodic response toward metoclopramide with a considerable enhancement (49-fold) compared to the bare GCE, thus was successfully applied for accurate determination of trace amounts of metoclopramide in pharmaceutical and clinical preparations (Shahrokhian et al., 2015)
Poly (diallyl dimethylammonium chloride)—CNC (PDDA–CNC) supported Au nanohybrids	Au nanoparticles	Biosensing	The 5Au/PDDA–CNCs (i.e., Au loading level of 5 wt%) exhibited the best glucose sensing ability with a low detection limit of 2.4 μM, high sensitivity of 62.8 μA mM^−1^ cm^−2^, and a linear detection range from 0.004 to 6.5 mM, which was ascribed to the moderate size and dispersity of the Au nanoparticles (Dong et al., 2016)
Polypyrrole - CNC (PPy-CNC) - based composite with glucose oxidase (GOx)	—	Biosensing	Prepared nanocomposite exhibited acceptable reproducibility, stability, and high sensitivity with high dynamic response ranging from 1.0 to 20 mM glucose
			Limit of detection (LOD) was (50 ± 10) µM and it also excluded interfering species, such as ascorbic acid, uric acid, and cholesterol (Esmaeili et al., 2015)
Bioprinting			
NFC—Alginate bioink	—	2D and 3D bioprinting	Shear thinning behavior of bioink enabled printing of both 2D grid-like structures as well as 3D constructs
			Anatomically shaped cartilage structures, such as a human ear and sheep meniscus, were 3D printed using MRI and CT images as blueprints
			Human chondrocytes bioprinted in the noncytotoxic, nanocellulose-based bioink exhibited a cell viability of 73 and 86% after 1 and 7 days of 3D culture, respectively (Markstedt et al., 2015)
Alginate sulfate—nanocellulose—bioink	—	Bioprinting	Non-printed bioink material promoted cell spreading, proliferation, and collagen II synthesis by encapsulated cells
			When bioink was printed, biological performance of the cells highly depended on nozzle geometry
			Cell spreading properties were maintained with the lowest extrusion pressure and shear stress (Müller et al., 2017)
NFC-alginate bioink	—	3D bioprinting	NFC-alginate bioink supported redifferentiation of human nasal chondrocytes while offering proper printability in a biologically relevant aqueous 3D environment, making it a promising tool for auricular cartilage tissue engineering and many other biomedical applications (Martínez Ávila et al., 2016)
NFC with alginate (NFC/A) and hyaluronic acid (NFC/HA)	—	3D bioprinting	Human-derived induced pluripotent stem cells were 3D bioprinted into cartilage mimics using NFC composite bioink for treatment of cartilage lesions
			Low proliferation and phenotypic changes away from pluripotency were seen in the case of NFC/HA.
			In the case of NFC/A constructs, pluripotency was initially maintained. After 5 weeks, hyaline-like cartilaginous tissue with collagen type II expression and lacking tumorigenic expression was observed. Also, a marked increase in cell number within the cartilaginous tissue was detected. NFC/A bioink is suitable for bioprinting iPSCs to support cartilage production in co-cultures with irradiated chondrocytes (Nguyen et al., 2017)

Another very common biomedical application of nanocellulose is in tissue engineering. The unique three-dimensional network formed by cellulose makes it an ideal candidate for a variety of tissue engineering applications. Other properties of nanocellulose, such as its mechanical strength and biocompatibility also add to its suitability for tissue engineering (Jorfi and Foster, 2015).

Tissue engineering is a novel field where cells, biomaterials, and growth factors are combined to produce engineered-organs and tissues for replacement of damaged tissues in the human body (Moroni et al., 2014). The required properties of scaffolds to be used for tissue engineering include good mechanical properties to sustain cell proliferation for more than 4 weeks, ability to support its differentiation into specialized structured tissues, and porous gel structure to allow gases transport and promote vascularization. The growth tissue, after sufficient development, is to be incorporated into the organism, expecting a minimal inflammatory response. Further, the scaffold should ideally degrade naturally into the body, without the need of any invasive procedure for its removal (Drury and Mooney, 2003). Nanocellulose fulfills all these requirements and thus is a suitable tissue engineering material (Curvello et al., 2019).

The most common type of tissue engineering application that nanocellulose is used for is wound healing. Chronic wounds pose an increasingly significant worldwide economic burden (over £1 billion per annum in the United Kingdom alone). With the escalation in global obesity and diabetes, chronic wounds will increasingly be a significant cause of morbidity and mortality (Jack et al., 2017). Wound dressing materials should have a porous network, ability to swell, a specific elasticity, and the ability to retain moisture and pH over time (Zhong et al., 2010). Since there always are high chances of infections, it is desired for dressings to also have antibacterial properties to minimize the bacterial growth during the healing process (Curvello et al., 2019). Nanocellulose has been extensively explored for scaffold and wound dressing applications. Nanocellulose is highly versatile and can be tailored with specific physical properties to produce an assortment of three-dimensional structures (hydrogels, aerogels, or films), for subsequent utilization as wound dressing material. It can also be loaded with antimicrobial agents to prevent infection in the wound (Kupnik et al., 2020).

After wound healing, bioprinting is another innovative tissue engineering strategy which nanocellulose is commonly used for. 3D bioprinting is emerging as a powerful tool for the construction of highly structured tissue engineering scaffolds. Through this technique, a 3D bioprinter is able to precisely dispense materials in three dimensions while moving in X, Y, and Z directions, enabling the production of complex structures from the bottom up (Markstedt et al., 2015). Cells are encapsulated within the printed material gel in homogeneous density and quantity. This feature makes 3D bioprinting better than the traditional two-step process where cells are inoculated into pre-made scaffolds, leading to heterogeneous cell distribution (Mandrycky et al., 2016).

It is often challenging to identify a bioink that supports cell growth, tissue maturation, and the successful formation of functional grafts to be used in regenerative medicine. In important research, a mitogenic hydrogel system based on alginate sulfate was identified as a bioink as it supports chondrocyte phenotype, but it was not printable due to its unfavorable rheological properties. To convert alginate sulfate into a printable bioink, the researchers combined it with nanocellulose, which has been shown to possess very good printability (Müller et al., 2017). The ability to spatially control the placement of cells, biomaterials, and biological molecules makes the nanocellulose a suitable tool for bioprinting (Martínez Ávila et al., 2016).

The important studies performed on biomedical applications of nanocellulose are summarized in Table 2.

## Legal Aspects of Botanical Nanocellulose

### EU Legislation

The free movement of goods, persons, services, and capital is part of the EU’s internal market. Free movement of chemicals is also related to the free movement of goods. Nanotechnologies and issues related to nanocellulose are of great importance and relevant legislation is also gaining importance. This gives rise to an obvious question, ‘how is the production and distribution of nanocellulose regulated and specifically what conditions must be satisfied in the case of nanocellulose from plants?’ The EU internal market could not function without regulation of chemical requirements. EU legislation in this area aims to ensure a high level of protection of human health and the environment (Rauscher et al., 2017). The protection of health and the environment is one of the prior areas of EU law, and EU legislation seeks to protect them.

EU legislation regulates registration of chemical substances. However, currently there is no specific EU legislation regulating nanocellulose, and therefore the general legislation of Regulation (EC) No 1907/2006 of the European Parliament and of the Council of 18 December 2006 concerning the Registration, Evaluation, Authorisation and Restriction of Chemicals (“REACH”) must be applied (Foth and Hayes, 2008; Kautto and Valve, 2019). It is therefore necessary to apply REACH to any handling of nanocellulose from plants. For REACH, the European Commission has repeatedly defined nanomaterials as a natural, incidental or manufactured material containing particles, in an unbound state or as an aggregate or as an agglomerate and where, for 50% or more of the particles in the number size distribution, one or more external dimensions is in the size range 1–100 nm. In specific cases and where warranted by concerns for the environment, health, safety, or competitiveness the number size distribution threshold of 50% may be replaced by a threshold between 1 and 50% (European Commission (2012)). However, the current legislative setting in EU law is not entirely perfect. The European Parliament has repeatedly called on the European Commission to revise REACH to better adapt to nanotechnologies, including nanocellulose from plants (European Commission (2012)).

### EU Legislation on the Registration of Chemicals

The basic legislation in the field of chemicals is REACH, establishing a European Chemicals Agency and Regulation (EC) No 1272/2008 of the European Parliament and of the Council of 16 December 2008 on classification, labelling, and packaging of substances and mixtures. Another regulation governing nanocellulose is Regulation (EU) No 649/2012 of the European Parliament and of the Council of 4 July 2012 concerning the export and import of hazardous chemicals Text with EEA relevance. Commission Regulation (EC) No. Regulation (EC) No 440/2008 laying down test methods pursuant to Regulation (EC) No 1907/2006 of the European Parliament and of the Council on the Registration, Evaluation, Authorization and Restriction of Chemicals (REACH). There is currently no legislation that targets nanotechnologies, including nanocellulose. Therefore, REACH can only be applied very generally.

REACH defines a substance within the meaning of Article 3 1) as follows: substance means a chemical element and its compounds in the natural state or obtained by any manufacturing process, including any additive necessary to preserve its stability and any impurity deriving from the process used, but excluding any solvent which may be separated without affecting the stability of the substance or changing its composition. Nanocellulose is a substance within the meaning of this article (Foth and Hayes, 2008). Under Article 6 1) of REACH, an application for registration of a substance is submitted to the Agency by any manufacturer or importer of a substance on its own or in one or more preparations in quantities of 1 tonne or more per year; the application is subject to a fee, the amount of which is set under REACH. The application for registration has the prescribed requirements which apply to the substance itself as well as to the manufacturer. The substance is then identified according to the sections in Annex VI of REACH (Article 7) and must be accompanied by a technical dossier and, in selected cases, a chemical safety report (Article 10 (a) and (b)). It also includes a requirement to prepare a chemical safety report for substances subject to registration in quantities of 10 tonnes or more per year (Article 14 (1)), unless an exemption within the meaning of Article 14 (2) is granted.

Not all substances are subject to registration as few are exempted due to their use for research and development. Substances that are manufactured or imported for research and development purposes are not subject to registration for 5 years (Article 9 (1)). However, even in such a case, the importer and the manufacturer are obliged to provide the Agency with sufficient information, such as the identification of the manufacturer and the substance (Article 9 (2)).

The application for registration is submitted to the Agency, which then checks all the details and assigns a registration number to the substance (Article 20 (1) and (3)). This is followed by an evaluation of the record, where the Agency reviews the testing proposals and then evaluates the substances (Article 44). The Agency then develops the criteria for prioritizing substances.

The Agency shall subsequently notify the registrants or users of the draft decision on the evaluation of the substance and shall inform them of the right to comment on that decision within 30 days of receipt of the proposal. The draft marketing authorization shall also be notified to the competent authorities of the Member States, including any comments from applicants. Member States then can also propose amendments to the decision (Article 50 (1) and Article 51 (1) and (2)).

Certain substances of very high concern that will be included in Annex XIV REACH require marketing authorization. Their placement on the market requires a justification as to why they must be used and whether they cannot be replaced by suitable alternatives or technologies; however, such implementation must be economically and technically feasible (Articles 55 and 56). These are, for example, carcinogenic or toxic substances. The European Commission then decides on the authorization of these substances (Article 60).

### Terms of Registration

REACH sets out the general conditions for the registration of a substance, i.e., nanocellulose in this case. The notification includes both the identification of the manufacturer or importer and, above all, the identification of the substance according to the Annex to REACH. Of particular importance is the technical record, which includes study summaries (Article 10 (a) (vi)). Nanocellulose may also be exempted from registration if it is for research and development (Article 9). However, even in the case of an exemption, it is necessary to identify the substance and duly justify its use for research and development.

### Reach and Nanocellulose

Nanocellulose from plants is a polymer that exists in nanoforms. It is a substance within the meaning of Article 3 (1) of REACH. This substance is subject to registration if more than 1 tonne per year is manufactured or imported into the EU. As these are nanoforms, the substance must be registered. However, if a substance is exempted under REACH, it would not be subject to registration. Conversely, if a substance meets the definition of nanoform in Annex VI of REACH and the substance is subject to registration, the nanoform condition must also be met. “Cellulose pulp” is exempted from registration in Annex IV of REACH; however, cellulose pulp is not nanocellulose. If they are naturally occurring substances and are not chemically modified, they shall also be exempted from registration under Annex V, provided it is not a dangerous substance.

### European Medicines Agency and Nanocellulose

The European Medicines Agency (EMA) is an agency of the European Union established by Regulation (EC) No 726/2004 of the European Parliament and of the Council of 31 March 2004 laying down community procedures for the authorization and supervision of medicinal products for human and veterinary use and establishing a European Medicines Agency. This regulation regulates three main areas: 1) the authorization and supervision of medicinal products for human use, 2) the authorization of veterinary medicinal products, and 3) the functioning of the European Medicines Agency. The EMA is responsible for coordinating the scientific resources and for the evaluation of medicinal products. The EMA must also cooperate with the Member States authorities and provide them the scientific knowledge about the specific effects of medicinal products. To achieve this goal, it creates, for example, a database of medicinal products (Articles 56 and 57).

No medicinal product, neither veterinary nor human, could be introduced in the EU market without authorization (Article 3). Registration applications are submitted to the EMA. Each application shall contain the particulars referred to in Article 6. The Agency shall decide on the registration in a specific period under Article 6 (3). The registration is then valid for a renewable period of 5 years (Article 14 1) and (2)). Marketing authorization is thus done by EMA. If the EMA carries out a marketing authorization, it is valid in all EU Member States and in Norway, Iceland, and Liechtenstein. The Regulation also addresses the issue of criminal and civil liability in such a way that the granting of a marketing authorization does not affect the civil and criminal liability of the manufacturer or marketing authorization holder (Article 15). The regulation also describes pharmacovigilance, which is crucial for placing on the internal market. The holder of a marketing authorization for a medicinal product for human use must always have at his disposal a qualified person responsible for pharmacovigilance (Article 23).

The EMA does not contain any specific regulation for nanocellulose. According to the EMA, however, nanocellulose from plants can be used as an excipient. Specific conditions then apply to excipients (Committee for Medicinal Products for Human Use, 2018). For example, the choice of this substance and other characteristics for determining the active substance must be explained. The EMA is related to REACH. If the excipient, including nanocellulose from plants, is a new substance then that would be subjected to the registration process under REACH. It therefore depends on the specific composition and use of nanocellulose from plants for therapeutic purposes. It can hence be concluded that if the botanical NC is to serve as a medicinal product, human or veterinary, it must be approved by the EMA. The registrant must then also submit the relevant dossiers to be assessed by the EMA.

## Future of Nanocellulose

In addition to renewability, better carbon footprint, sustainability, indispensability to bioeconomy, and environmentally benignity of nanocellulose, especially in comparison to petroleum-based products, the recent developments in science and technology have placed nanocellulose in the categories of future material of innovation and production. To understand the future scope of nanocellulose, we will consider different fields individually. In material science, nanocellulose based and/or reinforced composites for materials application in food packaging involving active packaging have been reported numerous times and some commercial products are already available. Active or smart packaging, which is a developing branch of food packaging technology, can be seen as a promising application of cellulose. The transparent paper and its applications in thin film transistors, organic light-emitting-diode, organic photovoltaic devices, printed foldable antenna, and resistive paper touch screen can give some idea of future applications of cellulose in high value areas. In the biomedical field, newly reported applications in tissue engineering, implants, prosthetics, drug delivery, patches, and wound healing using films, aerogels, and hydrogels, 3D printed structures add a very specific dimension to the high value applications of cellulose. Additionally, the already marketed bio-cellulose based products for tissue engineering, wound care, and wound healing are relatively new entrants to the catalog of cellulosic biomedical products. In light of the above arguments, the future of cellulosic materials in interdisciplinary and especially in high-value applications seems very bright and these materials can be assumed to be major contributors to future sustainable bio-economies at the global scale.

## Conclusion

Utilization of cellulose in human society is as old as utilization of paper. It has been a traditional product of the pulp and paper industry. Along with the development in the field of science, the understanding of structure and properties of cellulose has improved, which in turn has opened the doors for its newer applications. Better understanding of delignification process of wood and other lignocellulosic biomass has led to greener, economical, and environmentally favorable processes of cellulose production. Recent reports on biological compatibility and toxicity properties of cellulose have pulled cellulose from just being a tableting excipient and pH sensitive coating agent to being a drug carrier molecule and more. Cellulose is now being used in its nano form as nanocrystals and nanofibers, which are now extensively explored as drug carriers, tissue engineering material, in wound healing, 3D bioprinting, etc. A good number of pilot plant and semi-commercial scale demonstration and production units are already working across the globe and bodes well for the future of nanocellulose-based materials.

There is no specific legislative act in EU legislation regulating nanocellulose from plants. Thus, only the general REACH regulation, which regulates the manufacture, import, and registration of chemicals, can be applied. Several other regulations that follow the content of the REACH regulation will also be applied. For the registration of nanocellulose from plants, it will be necessary to submit the necessary documents for registration, in particular the exact identification of the substance. The condition of substance identification will also be necessary if nanocellulose from plants is exempted from registration for scientific and research purposes; however, this purpose must always be demonstrated by the importer or manufacturer.
